# A New Concept for Smart Drug Delivery: Adhesion Induced Nanovector Implosion§

**DOI:** 10.2174/1874104500802010062

**Published:** 2008-05-14

**Authors:** Nicola M Pugno

**Affiliations:** Department of Structural Engineering, Politecnico di Torino, Corso Duca degli Abruzzi 24, 10129, Torino, Italy; National Institute of Nuclear Physics, National Laboratories of Frascati, Via E. Fermi 40, 00044, Frascati, Italy

## Abstract

In this paper we show that controlling adhesion in highly flexible nanovectors can help in smartly delivering the drug. The high flexibility of the nanovector is used to smartly deliver the drug only at the target site by the new concept of “adhesion induced nanovector implosion”; a liquid drop analogy is developed for the calculations.

## 1 INTRODUCTION

Injectable drug-delivery nanovectors are used in nanomedicine for the cancer therapy, specifically for multiple drug delivery, thermal ablation or imaging, see the recent reviews [1,2]. These vectors must be large enough to evade the body defences but sufficiently small to avoid blockages of even the capillaries, thus nanosized by definition.

Nanovectors can extravasate into the tumor through the enhanced permeation and retention (EPR) effect [3], roughly schematized in Fig. (**1**). The increased permeability of the blood vessels, i.e. the formation of new vessels from existing ones, leads to the permeation of nanovectors into the tumor; moreover, its dysfunctional lymphatic drainage retains them, allowing a localized drug delivery. Experiments with liposomes suggest that extravasation into tumors can take place if their diameter is <400nm [4] and becomes more effective for size <200nm [4-7]. Unfortunately some tumors do no exhibit the EPR effect and this passive strategy could also induce a lack of control and thus multiple-drug resistance [2].

To overcome these limitations nanovectors are functionalized in order to actively bind to specific cells after extravasation, through ligand-receptor interactions. To maximize specificity, a surface marker (receptor or antibody) should be overexpressed on target cells relative to normal ones. It is generally accepted that higher binding affinity increases targeting efficacy. However, for solid tumors, it could reduce penetration due to a binding-site barrier, where the nanovectors bind to its target so strongly that penetration into the tissue is prevented [8,9]. This suggests the importance of optimizing adhesion in nanovectors that is the aim of the present paper. Moreover, cancer cells often overexpress the receptors for nutrition, thus growth factor or vitamin interactions with cancer cells is an additional commonly used targeting strategy.

Finally, the nanovectors are activated and release their cytotoxic action when irradiated by external energy or by the environmental conditions, such as metabolic markers or the acidity levels that accompany inflammatory states, infections and neoplastic processes [1].

Nanosized vectors include fusion proteins and immunotoxins/polymers (3-15nm), dendrimers (~5nm), polymer-drug conjugates (6-15nm), miscelles (5-100nm, lipid based or polymeric), nanoparticles (10-40nm, gold, for photothermal ablation; 50-200nm, polymeric), liposomes (85-100nm), polymersomes (~100nm), immunoliposomes (100-150nm) and nanoshells (gold-silica, ~130nm, photothermal therapy) [2]. Absorbing nanoshells are suitable for hyperthermia-based therapeutics, where they absorb radiation and heat up the surrounding cancer cells: above a thermal threshold irreversible damage selectively kills the cancer cells [2]. To achieve temporal release of two drugs, composite polymer core/lipid shell structures can be adopted [10].

Polymers are the most commonly explored materials for nanovectors. Lipid-based nanovectors have attractive biological properties, such as biocompatibility, biodegradability and isolation of drugs from the surrounding environment. Between them the most famous are liposomes, single- or multi-bilayered (within inner aqueous phases) spherical particles. They have shown preferential accumulation in tumor *via *EPR effect. However, too long-circulating liposomes may lead to extravasation of the drug into undesired sites. Long circulating half-life, soluble or colloidal behavior, high binding affinity, biocompatibility, easy functionalization, easy intracellular penetration, controlled pharmacokinetic and high drug protection are all characteristics simultaneously required for an optimal nanocarrier design.

However, controlling, under the body defences, both targeting and drug delivery remains a complex task. Mechanical studies can have a role in this, as suggested by the adhesion of colloids on a cell surface in competition for mobile receptors [11] or in receptor-mediated endocytosis [12]. In this note we will introduce a new concept, of smart highly flexible (a property that could be crucial for smart drug delivering but still ignored in the literature) nanovectors, based on smart adhesion [13,14]. Adhesion between a nanovector and a cellular substrate is governed by the adhesion energy (van der Waals, electrostatic, steric, etc…) per unit area and, due to the tremendous surface to volume ratio of nano-objects, becomes predominant at the nanoscale. Geckos and spiders take advantage of this [14], and nanovectors could make the same. During adhesion, the smart nanovector considerably changes its shape in a controllable way and, in case, can implode due to buckling [15]. Thus the high flexibility of the nanovector is used to release the drug only during adhesion, by the new concept of “adhesion induced nanovector implosion”; a liquid drop analogy [13] is used for the calculations. Such a mechanism smartly delivers the drug in a controllable way, ideally aborting the tumor colonization [16].

Obviously mechanical models in the field of life science have to be considered with caution, since much more complex mechanisms could be prevailing.

## 2 ADHESION OF LARGELY DEFORMED NANOVECTORS: A LIQUID DROP ANALOGY

The adhesion of liquid drops is a fascinating field, two hundred years old, see the noticeable review by Quèrè [17], and could present analogies with the adhesion of nanovectors [18], e.g. as recently observed for nanotubes [13]. For highly flexible nanovectors the inevitable presence of large displacements, deformations and contacts renders the problem out of the domain of linear elasticity and in that of the elastica theory of shells, for which only numerical integrations can be obtained, e.g. [15]. A liquid drop analogy could help in solving, even if in a approximated way, the problem: accordingly, we are going to extend the approach developed for cylindrical symmetry in [13] (applied to nanotubes) to the spherical symmetry (for nanovectors).

**Fig. (2):**
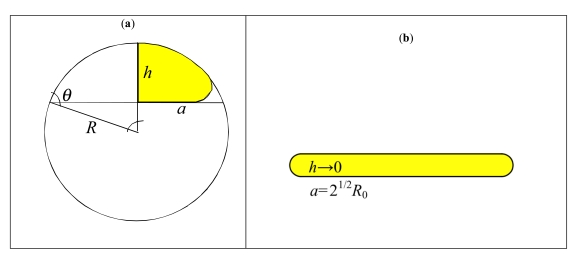
Nanovector (liquid drop) geometry, under large contact/deformation (**a**); squashed configuration (**b**).

The adhesion of a (small, for which surface tension prevails on gravity) liquid drop is fully described by the contact angle *θ* (a function of the liquid/solid/vapour surface energies) between drop and substrate, see Fig. (**2a**). Indicating with *R*_0_ the radius of the drop in air and with *R* the radius of curvature of the spherical cap describing the adhering drop, the radius of the contact area *a* can be calculated imposing the mass conservation. The adhesion of a nanovector of radius *R*_0_ can be similarly described by an equivalent contact angle *θ* (that we expect to be a function of the adhesion work and bending stiffness), the radius of curvature *R* of the deformed non-contact cap and the contact radius *a* = *R*sin*θ* ; thus the (maximum) height of the deformed nanovector is *h = R(1-cosθ)* , see Fig. (**2a**). Assuming a porous membrane of the nanovector, thus capable of exchanging mass and deliver drug, the surface inextensibility rather than the mass conservation has to be imposed, i.e. $S=2\pi \mathrm{Rh}+\pi a^{2}=4\pi R_{o}^{2}$ (Fig. **2a**). Accordingly we deduce:

(1) $$\frac{a}{R_{0}}=\frac{2}{\sqrt{1+2\left(1-\cos\theta \right)/\sin^{2}\theta }}$$

For small contacts *θ → π* and $a/R_{0}\approx \sqrt{2\sin^{2}\theta /\left(1-\cos\theta \right)}$. This asymptotic solution can be directly compared with the analytical result posed by elasticity for a spherical shell with Young’s modulus *E*, Poisson’s ratio *v*, thickness *t* and contact surface energy *γ*, which yields $a/R_{0}\approx \sqrt{2\sqrt{12}\pi R_{0}\gamma /\left\{{\mathrm{Et}}^{2}\right\}}$ [19]; thus, we can define the contact angle for a nanoshell (e.g. liposome) according to:

(2) $$\left\{\begin{array}{cc} \frac{\sin^{2}\theta }{1-\cos\theta }=\frac{R_{0}}{R_{0}^{\text{*}}} & R_{0}\leq {2R}_{0}^{\text{*}}\\ \frac{\sin^{2}\theta }{1-\cos\theta }=2 & R_{0}>{2R}_{0}^{\text{*}} \end{array};R_{0}^{\text{*}}=\frac{{\mathrm{Et}}^{2}}{\pi \sqrt{12}\gamma \left(1-\alpha \right)},\right.$$

in which the parameter 0 ≤*α* ≤1 takes into account the stiffening caused by the presence of a material (e.g. particles, liquid-like, etc.) inside the nanovector. For a porous membrane α ≈ 0 and the mass exchange has a vanishing energy cost. However, we may note that the membrane could become non-permeable because of the adhesion of the plasmatic proteins, unless proper wetting, electrostatic force, superficial charge are provided.

We have found that for a compact nanosphere eq. (2) would remain valid with $R_{0}^{*}=2R_{0}{\left\{2ER_{0}/\left(3\pi \gamma \right)\right\}}^{2/3}$.

According to eq. (2), the contact will be hydrophobic or better nanovector-phobic ( *θ* > *π*/2, water/nanovector repellent) or nanovector-philic ( *θ* < *π*/2) for $R_{0}<R_{0}^{\text{*}}$ or $R_{0}>R_{0}^{\text{*}}$; or super-nanovector-phobic/philic (*θ* ≈ *π*, 0 ) for *R_0_* → 0 or $R_{0}\geq R_{0}^{\left(C\right)}=2R_{0}^{\text{*}}$. For example, for a fullerene *E* ≈ 1TPa, *t* ≈ 0.34nm, *γ* ≈ 0.2J/m^2^ (C-C van der Waals), $R_{0}^{\text{*}}\approx 53\mathrm{nm}$ i.e. beyond a C1000000 molecule. A super-nanovector-phobic behavior results in a high motility, whereas a super-nanovector-philic behaviour is ideal for maximize adhesion. Note that eq. (2) implies that the contact area becomes maximal ( *θ* = 0) for $R_{0}\geq R_{0}^{\left(C\right)}=2R_{0}^{\text{*}}$ and thus not only for *R*_0_ → ∞ . This corresponds to a kind of implosion [15] (Fig. **2b**).

Introducing eq. (2) into eq. (1) we find the following nonlinear law:

(3) $$\left\{\begin{array}{c} \frac{a}{R_{0}}=\frac{2}{\sqrt{1+2R_{0}^{*}/R_{0}}}R_{0}\leq 2R_{0}^{*}\\ \frac{a}{R_{0}}=\sqrt{2}R_{0}>2R_{0}^{*} \end{array}\right.$$

For small contacts/deformations ( *θ* → *π* )  the prediction of eq. (3) is identical to the asymptotic solution reported in [19], whereas for large contacts/deformations ( *θ* → 0 ) ${a/R}_{0}=\sqrt{2}$, as coherently imposed by the inextensible condition (Fig. **2b**).

The (maximum) height of the flattened nanovector and its radius of curvature can be geometrically derived as (Fig. **2a**):

(4) $$\frac{h}{a}=\frac{1-\cos\theta }{\sin\theta },\frac{R}{a}=\frac{1}{\sin\theta }$$

For *θ* → 0 , *h/a* → 0 and * R/a* → ∞, where as for *θ* → *π*, * h*/(2*R*_0_) → 1 and * R/R*_0_ → 1 confirming that the theory is self-consistent.

This model is more applicable to nanoshells (liposome like) than other kind of nanoparticles proposed on the market (lipid, polymeric and up to dendrimers), for which it probably needs new hypotheses.

## 3 ADHESION INDUCED NANOVECTOR IMPLOSION

Designing a nanovector of size *R*_0_ with a proper elasticity, or $R_{0}^{\text{*}}$ see eq. (2), allow us to control the volume variation of the nanovector induced by the adhesion energy. If the nanovector membrane is perfectly porous ( *α* ≈ 0) an equivalent volume *ΔV* of drug (we assume here an ideal nanopump, thus a unitary efficiency) will be smartly and suddenly delivered only at the target. The nanovector works here as a nanopump. Geometrically we find:

(5) $$\Delta V=\frac{4}{3}\pi R_{0}^{3}-\frac{\pi }{3}\left(2+\cos\theta \right){\left(1-\cos\theta \right)}^{2}R^{3}$$

As a limit case for  $R_{0}=2R_{0}^{\text{*}}$ , i.e.*θ* = 0, $\Delta V=4\pi R_{0}^{3}/3$ and the nanovector is fully imploded as a consequence of the adhesion with the target. Diffusive slow mechanisms will release the remaining amount of drug, still contained in the deformed nanovector after adhesion. We could thus control separately fast and slow drug deliveries, in order to optimize drug efficiency, by realizing a two-stage temporal nanovector. As a limit case, the adhesion induced implosion of the nanovector can be required.

The nanopumping mechanism will be activated in addition to different delivering strategies, e.g. endocytosis, and could be used for a global optimal design. For example endocytosis is predicted with a maximal efficiency for particles having size *R* (2/*R* = 1/*R*_*p*1_ +1/R_*p*2_  for non-spherical geometry, in which 1/R_*p*1,2_ are the Gaussian principal curvatures) of ~20-30nm, with characteristic wrapping times of ~1-60s [12], even if other factors, in addition to the size, are expected to play a crucial role. An optimal colloid concentration also emerges [11]. We could imagine to delivery, by nanopumping, drug molecules with optimal size for endocytosis (two-stage drug delivering process). Nanopumping is expected to be basically immediate and thus faster than endocytosis, but a competition between these two mechanisms could take place.

## 4 CONCLUSIONS

In this paper we have shown that controlling adhesion in highly flexible nanovectors can help in smartly delivering the drug. The high flexibility of the nanovector is used to release the drug only during adhesion by nanopumping and, as a limit case, by the new concept of “adhesion induced nanovector implosion”; a liquid drop analogy have been used for the calculations, even if numerical exact solutions are under study. Fast (pumping) and slow (diffusion) drug deliveries can thus be separately controlled.

The author is supported by the ‘‘Bando Ricerca Scientifica Piemonte 2006’’ – BIADS: Novel biomaterials for intraoperative adjustable devices for fine tuning of prostheses shape and performance in surgery.

## Figures and Tables

**Fig. (1):**
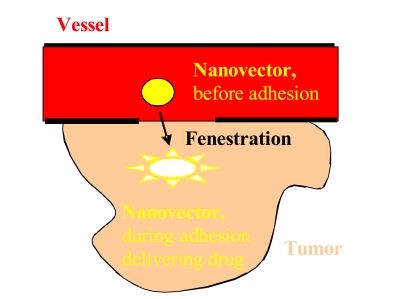
A simplified scheme describing the concept of the nanovector therapeutics.
